# Exchange-driven Magnetic Logic

**DOI:** 10.1038/s41598-017-12447-8

**Published:** 2017-09-22

**Authors:** Odysseas Zografos, Mauricio Manfrini, Adrien Vaysset, Bart Sorée, Florin Ciubotaru, Christoph Adelmann, Rudy Lauwereins, Praveen Raghavan, Iuliana P. Radu

**Affiliations:** 1 imec, Kapeldreef 75, B-3001 Leuven, Belgium; 2 KU Leuven, ESAT, B-3001 Leuven, Belgium; 3 Universiteit Antwerpen, Physics Department, B-2020 Antwerpen, Belgium

## Abstract

Direct exchange interaction allows spins to be magnetically ordered. Additionally, it can be an efficient manipulation pathway for low-powered spintronic logic devices. We present a novel logic scheme driven by exchange between two distinct regions in a composite magnetic layer containing a bistable canted magnetization configuration. By applying a magnetic field pulse to the input region, the magnetization state is propagated to the output via spin-to-spin interaction in which the output state is given by the magnetization orientation of the output region. The dependence of this scheme with input field conditions is extensively studied through a wide range of micromagnetic simulations. These results allow different logic operating modes to be extracted from the simulation results, and majority logic is successfully demonstrated.

## Introduction

The exploration and study of novel non-charge-based logic devices has been a main research focus in the past decade^1^. The purpose is to identify concepts that can extend the semiconductor industry roadmap beyond the complementary metal oxide semiconductor (CMOS) technology^1^. Since CMOS scaling, dictated by Moore’s Law^2^, will reach its limits in the following decade^3^, there is a need for logic components that can operate at high frequencies, be extremely compact and also consume ultra-low power^4^. A variety of magnetic devices have been benchmarked as promising candidates for low power applications^4–9^.

Among the most prominent concepts investigated for beyond-CMOS applications is the Nano-Magnetic Logic (NML) (also known as Magnetic Quantum Cellular Automata) that was first introduced by Cowburn *et al* ^10^. and Csaba *et al*.^11^. In NML, the information is encoded in the perpendicular magnetization (along +$\hat{{\bf{z}}}$ or −$\hat{{\bf{z}}}$) of ferromagnetic dots. The computation is mediated through dipolar coupling between nano-magnets. Although NML devices can be beneficial in terms of power consumption and non-volatility^4^, they have an operating frequency limited to about 3 MHz and an area around 200 nm × 200 nm^12^.

**Figure 1:**
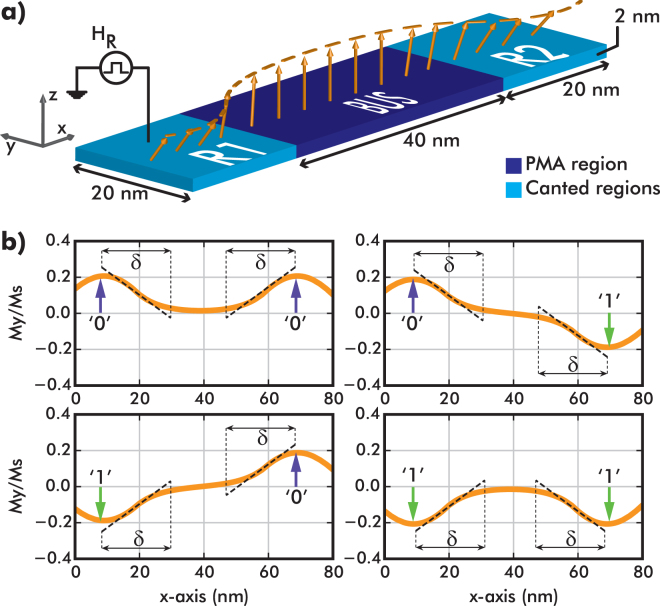
(**a**) The structure consists of an input (R1) and an output (R2) magnetic regions connected through a ferromagnetic bus. The length of the bus region is 40 nm. (**b**) Four possible initial magnetization states of the structure’s magnetization canted along the *y*-direction in the absence of any external field. The magnetization state of the R1 and R2 regions are represented by ‘0’ (if ${M}_{y}/{M}_{s}\simeq 0.2$) or ‘1’ (${M}_{y}/{M}_{s}\simeq -0.2$).

In this work, we propose a novel logic scheme that utilizes the direct exchange coupling as the main driver, in contrast to the NML concept. The structure, shown in Fig. 1a, is studied through a wide range of micromagnetic simulations. The device is 2 nm thick, 20 nm wide, and 80 nm long. It consists of a simple input/output (defined as R1 and R2, respectively) system that is interconnected through a magnetic bus. The magnetic anisotropy of the bus region is out-of-plane, while the R1/R2 regions have an in-plane anisotropy along $\hat{{\bf{y}}}$. Due to the exchange interaction, the magnetization is canted near the interfaces R1/bus and bus/R2. This canted region spans over a distance (*δ* - canting decay length) that depends on several parameters: the exchange constant *A*_*ex*_, the effective out-of-plane anisotropy of the bus *K*_*u*,*bus*_ and the in-plane anisotropy of the R1/R2 regions *K*_*u*_,_R1/R2_. When R1 and R2 have a length comparable to *δ*, they carry a bistable canted magnetization. Therefore, in this study, the R1/R2 length is set to 20 nm. Magnetic canted states have been used previously as spin wave generators and detectors in the Spin Wave Device concept^13^. However, in the proposed work, spin wave dynamics do not play any role in the operating principle, as the dominant interaction is the exchange interaction between the canted regions.

Interestingly, stable canted states have been observed by Gubbiotti *et al* ^14^. in Ni thin films. Exploiting the bistability of the canted magnetization regions, we can define four possible combinations of the R1/R2 states, depicted in Fig. 1b. The magnetization state of ${M}_{y}/{M}_{s}\simeq 0.2$ is defined to represent logic ‘0’ and the magnetization state of ${M}_{y}/{M}_{s}\simeq -0.2$ as the one to represent logic ‘1’. These four configurations can exist at equilibrium only if the bus is long enough to sufficiently decouple input and output. More specifically, if the distance between input and output were much smaller than 2*δ*, then the canted states would spread over the entire interconnect. As a consequence, the R1/R2 would become exchange coupled and the states 0/1 and 1/0 would not be allowed any more (for more details we refer the reader to the Supplementary Material). To avoid such a strong coupling, we set the interconnect length to 40 nm, which corresponds to ~2*δ*.

The input region (R1) is activated by the application of a local pulsed square-shaped magnetic field **H**_R_, which implements the external triggering of the structure. After the pulse, the state (0 or 1) is then transmitted to the output region (R2) via magnetic exchange interaction. This regional field (**H**_R_) is applied on the input in order to activate the logic operation of the magnetic structure and can be viewed as the equivalent of clocking signals in regular CMOS circuits. The field **H**_R_ can represent a physical excitation mechanism. For example, it can express an equivalent change of anisotropy and mimic electric field-controlled effects such as the magneto-electric^15^ or voltage-controlled magnetic anisotropy (VCMA)^16,17^ effects.

## Results

### Dynamic Behaviour

**Figure 2:**
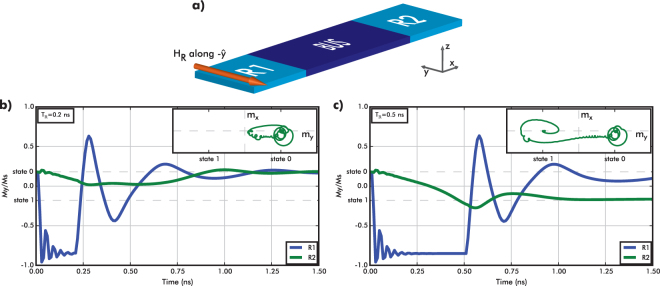
(**a**) Applied triggering field’s *H*_R_ = 0 direction. Magnetization dynamics of input (R1 - blue) and output (R2 - green) regions. Insets: projection of the output magnetization onto the *xy*-plane (**b**) when *T*_R_ = 0.2 ns: the output does not switch to state 1, (**c**) when *T*_R_ = 0.5 ns: the output does switch from state 0 to 1.

First, we investigate the impact of the input field conditions on the magnetization dynamics of the input and output regions. Figure 2 shows the normalized $\hat{{\bf{y}}}$-magnetization component averaged over the input and output regions for two simulations with equal input field (8 kA/m along −$\hat{{\bf{y}}}$, Fig. 2a) but different input field durations (*T*_R_ = 0.2 ns in Fig. 2b and *T*_R_ = 0.5 ns in Fig. 2c).

The magnetization of the input (blue curve) precesses and relaxes to a new equilibrium position, almost in-plane. Meanwhile, the output magnetization slowly transitions towards −$\hat{{\bf{y}}}$. When the input field duration is too short (Fig. 2b), the energy provided to the system is not sufficient for magnetization reversal, causing the output magnetization to oscillate back to its original state A. However, when the input field duration is increased to 0.5 ns (Fig. 2c), the output region magnetization smoothly transitions to state B. Comparing the insets in Fig. 2b and c, it is clear that the output magnetization switches when the field pulse is long (and large) enough to exceed the switching incubation time. In all simulations, we observe switching times that range from 0.5 ns to 0.75 ns, which are substantially faster than other spin-based technologies, such as NML^12^.

**Figure 3:**
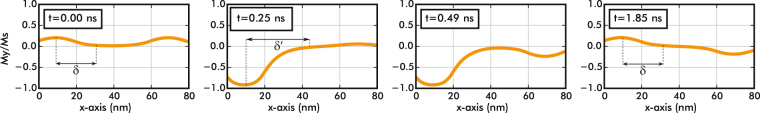
Evolution of My/Ms projected on $\hat{{\bf{x}}}$-axis over time. Input field appled is *H*_R_ = 8 kA/m and *T*_R_ = 0.5 ns.

In Fig. 1, at *H*_R_ = 0, states 0 and 1 are allowed for both R1 and R2 regions as they constitute stable magnetization configurations that are not interacting with each other because the canting decay lengths (*δ*) are not overlapping. This means that R1/R2 are exchange decoupled. In contrast, in the simulations of Fig. 2, the input magnetization reaches an angle that is beyond state B, due to the applied field. As a consequence, the canted input region becomes wider, as shown in Fig. 3. In particular, the canting decay length (*δ*′) spreads beyond the middle of the bus and advances onto the output canted region. Due to the magnetic exchange interaction, the output magnetization starts to move toward −$\hat{{\bf{y}}}$ and can finally switch.

In summary, the larger deviation angle at the input induces a broadening of the canted region that causes an exchange torque on the output. This torque enables the switching. Consequently, this scheme works if the interconnect is long enough for ‘exchange decoupling’ of R1/R2 at *H*_R_ = 0, and if it is short enough for exchange coupling of R1/R2 above a critical value of *H*_R_ at the input.

### Switching Mechanism

**Figure 4:**
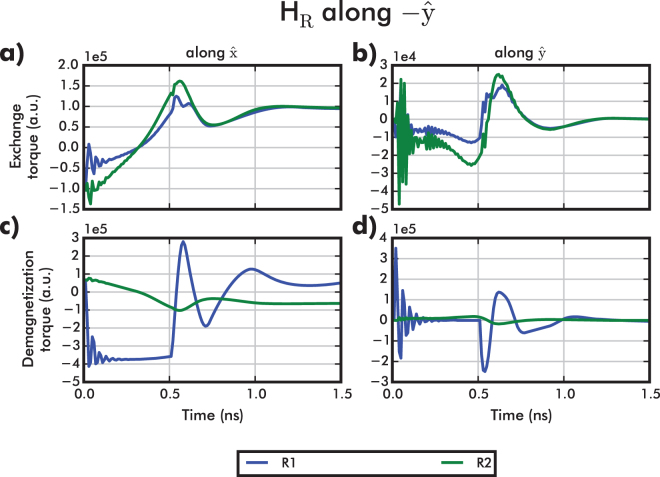
Acting torques on input (R1) and output (R2) regions as a function of time, calculated by Eq. 1. (**a**) $\hat{{\bf{x}}}$-component of the exchange torque. (**b**) $\hat{{\bf{y}}}$-component of the exchange torque. (**c**) $\hat{{\bf{x}}}$ -component of the demagnetization torque. (**d**) $\hat{{\bf{y}}}$-component of the demagnetization torque.

The switching mechanism driven by exchange coupling is verified in Fig. 4. Accounting for each contribution to the total effective field, we calculate the corresponding torques in both input and output regions. The torque component for a specific contribution is given, in arbitrary units (a.u.), by:

1 $${\tau }_{i}^{\beta }(t)=-{m}_{{\rm{R}}\mathrm{1/}R2}(t)\times {{\bf{H}}}_{\beta }(t)\cdot {\hat{e}}_{i}\mathrm{.}$$

where ${{\bf{m}}}_{{\rm{R}}\mathrm{1/}R2}(t)$ is the magnetization of either the input or the output region, *β* denotes the field contribution (e.g. exchange) and ${\hat{e}}_{i}$ the unit vector (i.e. ${\hat{e}}_{i}\in [\hat{{\bf{x}}},\hat{{\bf{y}}},\hat{{\bf{z}}}]$).

Figure 4a–d show the dynamics of the exchange and demagnetization torques along $\hat{{\bf{x}}}$ and $\hat{{\bf{y}}}$. The anisotropy field and the $\hat{{\bf{z}}}$ components are not shown because their relative effect is negligible. We observe that the magnitude of the exchange torque exerted on the output magnetization (green lines) is much larger than the dipolar torque. The exchange-driven mechanism for output switching is therefore confirmed. Moreover, note that the exchange torques exerted on the input and the output are strongly correlated (Fig. 4a and b), whereas the dipolar torques are very different. This confirms that the coupling between input and output is mainly due to the exchange interaction.

### Logic Operation

In order to implement logic with the exchange-driven scheme, we need to extract the logic behavior of the structure from the micromagnetic simulations. For this it is useful to be able to easily represent the information (magnetic canted states) in the system. We represent an unknown logic state with ‘X’ and the logic state of the entire structure with a pair like ‘01’, where the first letter represents the state of region R1 and the latter the state of R2. In order for the R1/R2 structure to perform logic, we should be able to identify at least two logic operating modes:

M1) XX $\mathop{\longrightarrow }\limits^{{H}_{{\rm{R}}}}$X0 or XX $\mathop{\longrightarrow }\limits^{{H}_{{\rm{R}}}}$X1: the output is set to state 0 or M1 after applying a triggering field. Mode M1 corresponds to state initialization.

M2) 0X $\mathop{\longrightarrow }\limits^{{H}_{{\rm{R}}}}$X0 or 1X $\mathop{\longrightarrow }\limits^{{H}_{{\rm{R}}}}$X1: the output is set to the initial input state (0 or 1) after applying a triggering field. Mode M2 corresponds to state propagation.

**Figure 5:**
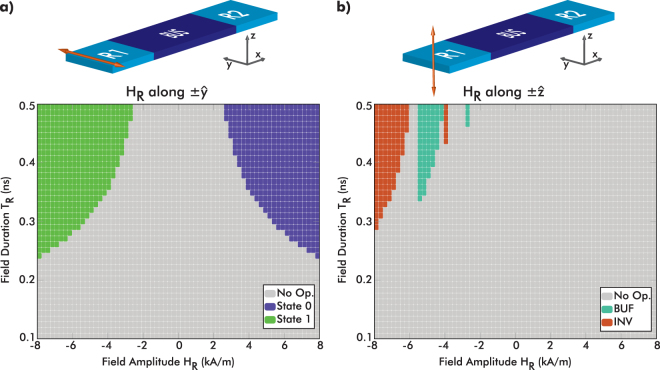
(**a**) Phase diagram of the operating regions (i.e. mode M1) as a function of input amplitude and duration. The input field is applied along the *y*-axis. (**b**) Phase diagram of the operating regions (when the output switches) versus the input amplitude and duration when input is applied along-$\hat{{\bf{z}}}$. Inverter mode (orange) represents the cases (0X $\mathop{\longrightarrow }\limits^{{H}_{{\rm{R}}}}$X1, 1X $\mathop{\longrightarrow }\limits^{{H}_{{\rm{R}}}}$X0). Buffer mode (cyan) represents the cases (0X $\mathop{\longrightarrow }\limits^{{H}_{{\rm{R}}}}$X0, 1X $\mathop{\longrightarrow }\limits^{{H}_{{\rm{R}}}}$X1).

We sum up all input field conditions to extract the operating regions that result in one of the desired modes (M1, M2). The simulations satisfying the logic operating mode M1 are shown in color in Fig. 5a.

The output state 0 (purple) is triggered only when **H**_R_ is applied along +$\hat{{\bf{y}}}$, whereas the output state 1 is set up only when **H**_R_ is applied along −$\hat{{\bf{y}}}$. Interestingly, output 0 or output 1 can be chosen by changing only the field direction while keeping the same field amplitude. From this duality, one can define a logic zero (input along +$\hat{{\bf{y}}}$→ state 0) and a logic one value (input along −$\hat{{\bf{y}}}$ → state 1). The second logic mode M2 corresponding to state propagation, is identified in the phase diagram of Fig. 5b. When applying an input field pulse along $\hat{{\bf{z}}}$, the output magnetization state correlates with the input magnetization in two ways: the blue region in Fig. 5b denotes the normal state propagation (buffer mode, as expressed in M2) and the orange regions denote the inverted state propagation (inverter mode), meaning all the 0X $\mathop{\longrightarrow }\limits^{{H}_{{\rm{R}}}}$X1 or 1X $\mathop{\longrightarrow }\limits^{{H}_{{\rm{R}}}}$X0 transitions.

Two important facts are deduced from this phase diagram. First, the buffering mode (cyan) allows the definition of an input field that can be used for clocking and is different from the values extracted for logic ‘0’ and ‘1’ in M1. Second, is the reconfigurability of this logic structure. If the input field amplitude changes, the operating mode can be switched between buffer (BUF) and inverter (INV). Moreover, this reconfigurability has a relatively large input duration ‘jitter’ margin (~0.15 ns), where any duration between 0.35 ns and 0.5 ns will result in the same operation for specific input field amplitudes.

### Exchange-driven Majority Gate

**Figure 6:**
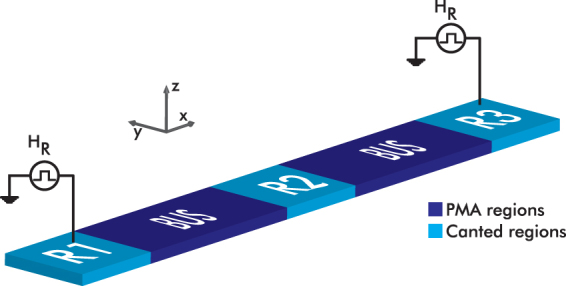
Majority gate structure of exchange-driven magnetic logic. The initial canted magnetization states of regions R1, R2, R3 are considered the three inputs of the gate. Two triggering magnetic pulses are applied at regions R1 and R3 and the sum of all three contributions result in R2 having the result of the gate.

To actually exploit this concept in logic applications only a majority gate is needed in addition to the buffer and inverter components^18^. Majority-based logic has been proven to be very beneficial for many Beyond-CMOS technologies^19^. The majority (MAJ) gate implementation is shown in Fig. 6, where a third canted magnetization region has been added to the initial structure of Fig. 1. The magnetization states of these three regions are considered as the gate’s inputs and the output is defined as the final state of the middle region after the application of the triggering magnetic pulses. This means that in this scheme region R2 serves both as an input and as an output.

Based on this structure, we can define the majority operating mode, similar to M1 and M2 as:

M3) XXX $\mathop{\longrightarrow }\limits^{{H}_{{\rm{R}}}}$ XMX: where M is the majority of the three initial states of R1, R2, R3 (e.g. 001 $\mathop{\longrightarrow }\limits^{{H}_{{\rm{R}}}}$X0X).

**Figure 7:**
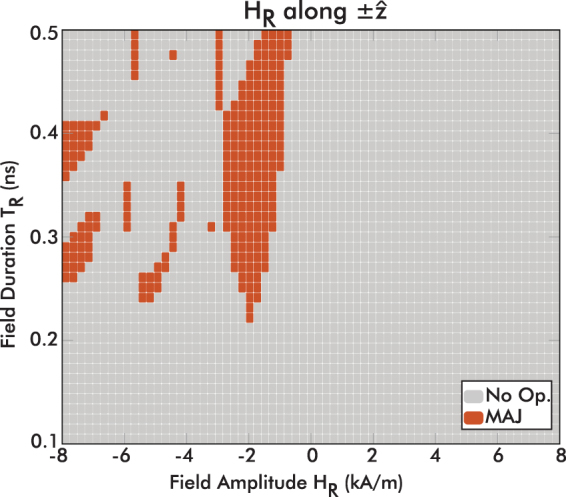
Phase diagram of the Majority gate operating regions (when the R2 regions switches to the correct majority result) of the input amplitude and duration, when fields are applied along $\hat{{\bf{z}}}$. In the majority gate two triggering fields are applied and both are swept simultaneously.

The only difference of this operation in the exchange-driven logic scheme is that two triggering fields are applied instead of one. These fields in regions R1 and R3 push the canted magnetization states out of equilibrium and in turn both apply an exchange torque on the middle region (R2). The final magnetization state of region R2 depends on the sum of R1 and R3 contributions through torque, as well as on the initial state of R2. The phase diagram of this structure is shown in Fig. 7.

In Fig. 7, we show the results of micromagnetic simulations varying the amplitude and duration of both magnetic field pulses applied (simultaneously) on regions R1 and R3. The operating regions of the majority gate are scattered because the interaction of three canted magnetization structures is complex and quite sensitive to variations. Interestingly, these operating regions partially overlap with the BUF/INV operating regions shown in Fig. 5c. By applying the exact same triggering field conditions we can achieve a BUF/INV or a majority gate operation depending on the device design.

### Logic circuit implementation

**Figure 8:**
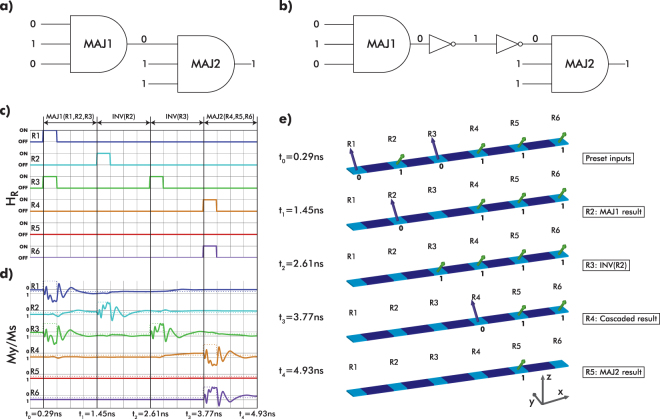
(**a**) Example circuit with two cascaded majority gates. (**b**) Translated circuit to accommodate the exchange-driven logic structures. (**c**) Triggering field pulses applied at each region (R1 to R6) of the structure to implement either the majority or inverter operation. (**d**) Normalized magnetization along $\hat{{\bf{y}}}$-axis over time for each of the regions of the structures. ${M}_{y}/{M}_{s}\simeq 0.2$ is represented by ‘0’ and ${M}_{y}/{M}_{s}\simeq -0.2$ is represented by ‘1’. (**e**) Schematic showing the magnetization states of the structure at different time points of the entire circuit operation.

In order to showcase the flexibility of the exchange-driven logic concept, we select the fastest field conditions that satisfy the INV/MAJ operations. These conditions are *H*_R_ = 8 kA/m and *T*_R_ = 0.29 ns. With this triggering field we can implement any logic circuit, an example of which is shown in Fig. 8a.

Since the output of the exchange-driven majority gate is situated in the middle region (R2 in Fig. 6), it is necessary to use two more circuit elements (inverters) that will allow the output result to be cascaded. This equivalent circuit is shown in Fig. 8b and consists of two majority gates (MAJ1 and MAJ2), cascaded with the use of two inverters. The exchange-driven logic structure that can implement the circuit of Fig. 8a is simply a straight line structure shown in Fig. 8e. The first three canted regions of the structure (R1, R2, R3) are the equivalent of the gates MAJ1 and the first INV of Fig. 8b. The other three regions (R4, R5, R6) implement the second majority gate (MAJ2) and the link between R3 and R4 implement the second INV of Fig. 8b. The values of the input signals of the circuit are shown in Fig. 8a and are assumed to be preset in the canted magnetization states of the exchange-driven logic structure (Fig. 8e). Figure 8c shows the triggering fields applied to each region of the exchange-driven logic over time and which actual circuit element is implemented. The duration of each pulse is *T*_R_ = 0.29 ns and the difference between two field applications is 3 · *T*_R_ to allow the magnetization state to settle in the desired stable state. The amplitude of all the pulses is *H*_R_ = 8 kA/m. The magnetization behavior of the regions is shown in Fig. 8d, where ${M}_{y}/{M}_{s}\simeq 0.2$ is represented by ‘0’ and ${M}_{y}/{M}_{s}\simeq -0.2$ is represented by ‘1’. Finally in Fig. 8e, the magnetization states of the whole structure are shown over time. We can see that at each snapshot of the operation the magnetization states contain a correct result of the logic operation. For example, at time *t*_1_ the R2 state is equal to the result of the MAJ1 operation and at time *t*_3_ the state of R4 contains this propagated result.

With this example, we show that the exchange-driven logic concept is amenable to cascading, that the operation of both an INV and a MAJ have equal delay (4 · *T*_R_ = 1.16 ns), and that two stages of majority gates can be operated under 5 ns. These two delays are relatively low compared to the majority of other magnetic logic schemes^4,12^.

### Physical realization

**Figure 9:**
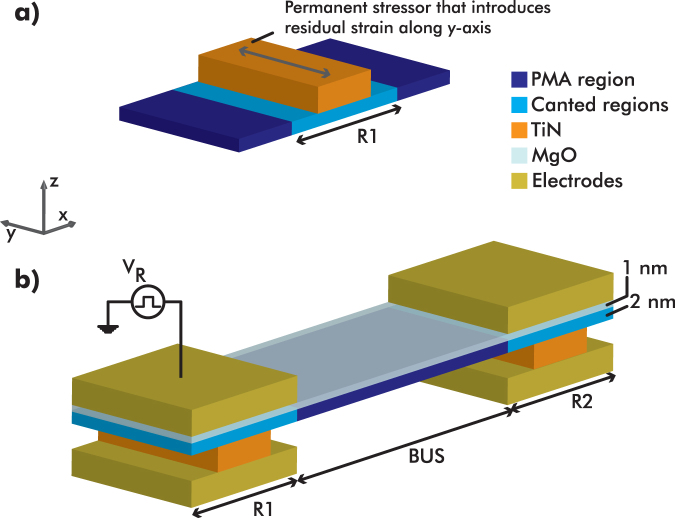
(**a**) Schematic of rectangular TiN layer that introduces residual strain along the $\hat{{\bf{y}}}$-axis. (**b**) Schematic of material stack that allows application of triggering voltages and integration with other CMOS components.

The principle of operation of exchange-driven magnetic logic relies on two key points: the magnetization canting in R1/R2 and the exchange-driven switching of these canted regions. These two ingredients are combined together to perform Boolean computation in a novel fashion. Figure 9 presents an overview of the proposed physical realization of both a canted magnetization region and a field application scheme to activate exchange-driven interaction between two regions.

To realize the bistable canted states of selected regions (such as R1, R2 etc.), we assume that all magnetic regions canted and bus are made of the same Co/Ni multilayers, with the same number of repetitions and the same thickness (2 nm). Such a material stack is known to be perpendicularly magnetized^20^. Therefore, two more steps have to be performed: first, cancel the out-of-plane anisotropy of the canted regions; second, create an in-plane anisotropy along the $\hat{{\bf{y}}}$-axis in these regions. These two steps are achieved as follows:

1. Selective ion bombardment of R1/R2 regions to damage the out-of-plane anisotropy^21^. Experimental details on the impact on a Co/Ni multilayer stack can be found in the supplementary material.
2. Creation of strain-induced anisotropy in R1/R2 by depositing TiN on top (see Fig. 9a). Due to its residual strain this additional layer acts locally as a permanent stressor^22^. Due to the magnetostriction of Co/Ni, a weak in-plane anisotropy is induced, resulting in canted magnetization along ±$\hat{{\bf{y}}}$.

Based on Gómez *et al*.^22^, TiN residual stress levels can easily exceed *σ*_res_ = 1 GPa. Given that the Young’s modulus of Co and Ni are 200 GPa^23^, the residual strain applied on the Co/Ni multilayer is *ε*_res_ = 0.5%. The anisotropy induced by the residual strain of TiN and Co/Ni magnetostriction is given by ref.^8^:

2 $${K}_{{\rm{res}}}=\frac{3}{2}\cdot {\lambda }_{{\rm{Co}}/{\rm{Ni}}}\cdot {Y}_{{\rm{Co}}/{\rm{Ni}}}\cdot {\varepsilon }_{{\rm{res}}}\mathrm{.}$$

assuming that the magnetostrictive constant of Co/Ni is ${\lambda }_{\mathrm{Co}/\mathrm{Ni}}=50\,ppm$ then ${K}_{{\rm{res}}}=75\,kJ/{m}^{3} > {K}_{u,R\mathrm{1/}R2}$. Hence, a TiN permanent stressor can induce enough in-plane anisotropy (relatively weak, compared to the out-of-plane anisotropy of the bus region - see Table 2).

A common advantage that most spin/magnetization-based logic concepts share is their material compatibility with the existing CMOS technology^24,25^. Since the proposed concept requires external triggering signals to activate the logic operations (*H*_R_ field pulses), these signals have to be controlled and distributed. Exploiting the material compatibility, the exchange-driven magnetic logic can be integrated together with the CMOS control logic and interconnections in a three-dimensional manner. Figure 9b shows how the exchange-driven magnetic logic concept can be isolated, using a thin layer of MgO (1 nm), to enable voltage pulse application that will excite the required magnetic field pulses (*H*_R_). Additionally, this scheme is CMOS integration compatible, as thin MgO layers for isolation have been used in conjunction with Co/Ni multilayers in spin-transfer torque magnetic random access memory (STT-MRAM) stacks^26^.

Based on the scheme shown in Fig. 9b, we can calculate a first-order energy consumption for such structure. The baseline energy consumption for a two-region structure (BUF/INV), will be the energy required to charge the capacitor of a region R. This energy is given by:

3 $${U}_{{\rm{R}}}=\frac{1}{2}\cdot C\cdot {V}_{{\rm{R}}}^{2}=\frac{1}{2}\cdot \frac{A\varepsilon {\varepsilon }_{0}}{d}\cdot {({E}_{{\rm{R}}}d)}^{2}\mathrm{.}$$

where *A* = 20 *nm* × 20 *nm* is the area of the capacitor, *ε* = 9.83 is the dielectric constant of MgO^27^, *d* = 1 *nm* is the thickness of the MgO layer (which represents the dielectric of the capacitor, since the Co/Ni multilayers of a region R are also conductive), and *E*_R_ is the electric field penetrating the MgO layer. Based on Shiota *et al*.^17,28^ the electric field (*E*_R_) required to generate a magnetic field of H_R_ = 8 kA/m is approximately ${E}_{{\rm{R}}}\simeq 0.15\,V/nm$, which would correspond to a triggering voltage ${V}_{{\rm{R}}}\simeq 0.15\,V$.

Given the aforementioned values, Eq. 3 is calculated:

4 $${U}_{{\rm{R}}}=0.395\,aJ\mathrm{.}$$

To calculate energy consumption of the INV and MAJ gates we have to multiply *U*_R_ by the number of inputs and triggered regions of each gate. Thus, we calculate ${U}_{{\rm{INV}}}=2\cdot {U}_{{\rm{R}}}$ for the INV gate (1 input, 1 region) and ${U}_{{\rm{MAJ}}}=5\cdot {U}_{{\rm{R}}}$ for the MAJ gate (3 inputs, 2 regions). In Table 1 we compare the aforementioned consumption with their equivalent ones in CMOS technologies and we highlight the low energy consumption potential of exchange-driven magnetic logic.

**Table 1:** Energy consumption per operation for INV and MAJ gates in exchange-driven magnetic logic and three CMOS technology nodes.

	This work	7 nm node^29^	15 nm node^30^	45 nm node^31^
INV	0.790 aJ	81 aJ	560 aJ	1.91 fJ
MAJ	1.975 aJ	560 aJ*	2.8 fJ**	7.81 fJ

*Calculated as the energy sum of 3 NAND2 gates and a NOR3 gate.

**Calculated as the energy sum of a INV gate and a MUX2 gate^32^.

Compared to latest state-of-the-art CMOS technologies, the exchange-driven magnetic logic consumes 100× lower energy consumption of an INV gate and 280× lower energy consumption of a MAJ gate.

## Discussion

We have presented a novel logic scheme based on bistable canted magnetization states. Given that the calculated operating energy is low (1.975 aJ), we can project that this logic scheme can enable implementation of low energy consumption logic circuits. This system also enables successful cascadable majority-based logic, a key component for bringing spintronic logic concepts to manufacturing. The underlying mechanism reveals that magnetic exchange interaction is the dominant driving force, enabling advanced and ultra-scaled logic components in the sub 30 nm regime (inverter area: 20 nm × 80 nm). Moreover, it proves to be very fast (one operation in 1.16 ns) compared to other spin-based logic schemes^6,12^. To further improve the operating windows and applicability for this logic scheme, materials have to be configured in such a way that they enable higher canted state angles. Additionally, this concept should be extended to a two-dimensional grid. A system-level overview that includes the required CMOS overhead in terms of energy consumption and delay has to be defined. Finally, based on the sequential nature of the concept it would be a candidate for wave-pipelining performance boosting^33^.

## Methods

We performed micromagnetic simulations of this structure using the micromagnetic solver OOMMF^34^. Moreover, the Runge-Kutta-Fehlberg method as derived by Dormand and Prince^35,36^ was used to integrate the Landau-Lifshitz-Gilbert (LLG) equation^37,38^:

5 $$\frac{\partial {\bf{m}}}{\partial t}=-{\gamma }_{0}\,{\bf{m}}\times {\bf{H}}+\alpha ({\bf{m}}\times \frac{\partial {\bf{m}}}{\partial t})$$

The mesh cell size is 2 nm × 2 nm × 2 nm and the bus is extended before and after the R1/R2 regions to allow for magnetization relaxation and avoid edge reflections. Thus, the simulated structure represents an R1/R2 arrangement on an infinitely long bus. For more details on this structure, please refer to the supplementary material. The material properties and input field parameters are presented in Table 2.

**Table 2:** Micromagnetic simulation material properties and input field values.

	Parameter	Value
*Materials	Exchange constant (*A*_*ex*_)	0.2 pJ/m
	Saturation magnetization (*M*_*s*_)	500 kA/m
	Bus region anisotropy (*K*_*u*,*bus*_)*	500 kJ/m^3^ (along $\hat{{\bf{z}}}$)
	Bus region damping	0.02
	R1/R2 region anisotropy (*K*_*u*_,_R1/R2_)	65.2 kJ/m^3^ (along $\hat{{\bf{y}}}$)
	R1/R2 region damping	0.2
*Input	Amplitude (*H*_R_) sweep	[0.5:0.25:8.0] kA/m
	Duration (*T*_R_) sweep	[0.1:0.01:0.5] ns
	Direction sweep	[±$\hat{{\bf{x}}}$, ±$\hat{{\bf{y}}}$, ±$\hat{{\bf{z}}}$]

*Defined as ${K}_{u,bus}=\frac{{K}_{s}}{t}$, surface anisotropy over thickness.

The saturation magnetization *M*_*s*_, the exchange constant *A*_*ex*_ and anisotropy constants (*K*_*u*,*bus*_, *K*_*u*,,*R*1/*R*2_) are within range for Co/Ni multilayers^39^. Additionally, the absence of noble metals makes them the right CMOS-compatible solution. The R1/R2 region anisotropy *K*_*u*,,*R*1/*R*2_ is explicitly considered to have a low value to allow the creation of canted magnetization with a small angle ${\theta }_{R\mathrm{1/}R2}\simeq {10}^{\circ }$ with respect to the perpendicular axis. The damping of the R1/R2 regions was set to a high value (0.2) to enable quick stabilization of the magnetization without many oscillations. Such high value can be achieved through magnetic stack engineering. To investigate thoroughly the behavior of the structure, the input field amplitude *H*_R_ and pulse duration *T*_R_ were varied with a fine granularity and the magnetization and field dynamics were monitored over the entire system.

## Electronic supplementary material

Exchange-driven Magnetic Logic - Supplementary Material
