# Supplementary material for: Exchange-driven Magnetic Logic
Source: Sci Rep. 2017 Sep 22;7:12154. doi: 10.1038/s41598-017-12447-8 (PMC5610253; doi:10.1038/s41598-017-12447-8)
Supplement: Supplementary file 1 — Exchange-driven Magnetic Logic - Supplementary Material [file 41598_2017_12447_MOESM1_ESM.pdf]

Exchange-driven Magnetic Logic - Supplementary Material

Odysseas Zografos[1,2,*], Mauricio Manfrini[1,+], Adrien Vaysset[1,+], Bart Sorée[1,2,3], Florin Ciubotaru[1], Christoph Adelmann[1], Rudy Lauwereins[1,2], Praveen Raghavan[1], and Iuliana P. Radu[1]

[1]imec, Kapeldreef 75, B-3001 Leuven, Belgium
[2]KU Leuven, ESAT, B-3001 Leuven, Belgium
[3]Universiteit Antwerpen, Physics Department, B-2020 Antwerpen, Belgium
[*]Odysseas.Zografos@imec.be
[+]these authors contributed equally to this work

ABSTRACT

Supplementary material containing details about the magnetization behavior and the simulation structure.

R1/R2 coupling

In the main text, it is shown that the structure operates when the distance between the R1 and R2 regions is L_{12}=40 nm. This choice is made so that the input and output are not completely coupled by exchange interaction and the magnetization of the two regions can be stabilized in different canted states. Fig. 1 shows the $\hat{\mathbf{y}}$-axis magnetization of R1/R2 regions when the distance between them is L_{12}=20 nm and a triggering field of H_R=8 kA/m and T_R=0.5 ns is applied along -$\hat{\mathbf{y}}$.

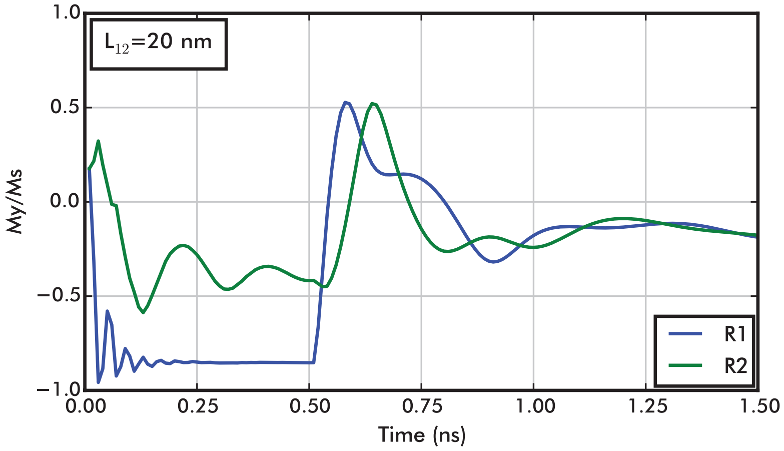

Figure 1. Magnetization dynamics of R1/R2 regions when L_{12}=20 nm.

It is clear from Fig. 1 that the magnetizations of the R1 and R2 regions are coupled, which means that the R2 magnetization always follows the magnetization of the R1 region. This happens for all triggering field conditions simulated, which means that having an L_{12} of 20 nm is not useful in terms of creating logic.

Simulation structure damping

To avoid edge effects and reflections near the region of interest in the simulation structure (R1/R2 regions and bus) we have extended the structure with 200 nm before and after the R1/R2 regions as shown in Fig. 2.

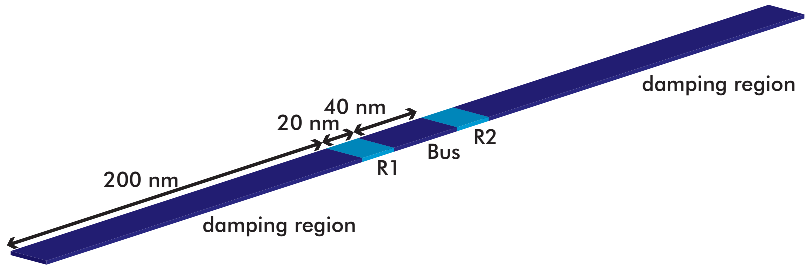

Figure 2. Complete view of simulation structure.

In order to ensure that any oscillations or spin waves propagating in the extended regions will be damped out, the damping of the structure is defined by:

$$\alpha(x) = \begin{cases} 0.2 & \text{if } x \in \mathrm{R1/R2} \\ 1.02 + \tanh\left(\frac{|x-\mu|-\rho}{\sigma}\right) & \text{otherwise} \end{cases} \quad (1)$$

where $x \in [0 - 4.8]\cdot 10^{-7}$ m, μ=2.4$\cdot$10^{-7}, ρ=2$\cdot$10^{-7} and σ=0.1$\cdot$10^{-7}.

Fig. 3 depicts the damping defined in Eq. 1. With this profile the R1/R2 structure acts as if it was placed in an infinitely long magnetic stripe and the behavior extracted from the simulations is free of any edge artifacts.

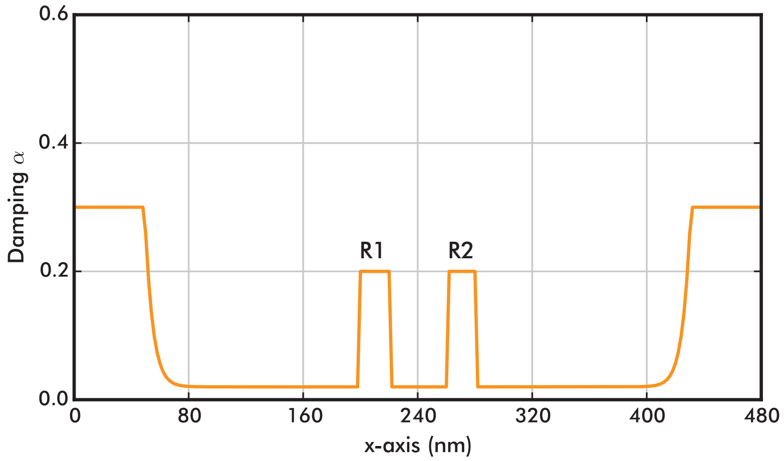

Figure 3. Damping of complete structure to absorb residual spin oscillations.

Ion bombardment of Co/Ni multilayer

One of the most critical parts of our proposed concept is the selective transformation of the Co/Ni multilayer structure from out-of-plane magnetization to in-plane. In order to demonstrate this, we first prepared Co/Ni magnetic multilayers by DC-sputtering with the following composition: TaN(1)/Ta(2)/[Ni(0.6)/Co(0.3)]4/Ni(0.6)/Co(0.6)/Ru(2)/Ta(2), layer thicknesses in parentheses are in nm, shown in Fig. 4a. Fig. 4b shows M(H) hysteresis loops for samples submitted to Xe ion bombardment treatment at various times t.[1] These loops were recorded with vibrating sample magnetometer (VSM) at room temperature. The saturation magnetization Ms of as-deposited samples (t=0 s) is about 650 emu/cc with very clear signature of PMA. With increasing time, Ms decreases to about 350 emu/cc for t=40 s showing completely IMA.[2] This is the first in creating a bistable canted state required for the R1/R2 regions.

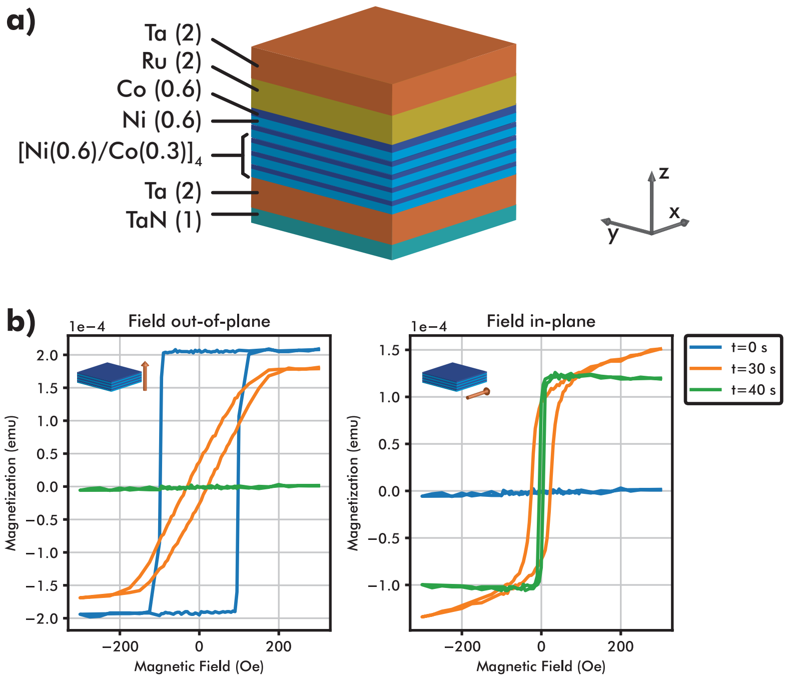

Figure 4. (a) Material stack of ion bombardment experiments, layer thicknesses in parentheses are in nm. (b) VSM measurements when applied external field is out-of-plane and in-plane respectively.

References

1. Franken, J.H. *et al.* Precise control of domain wall injection and pinning using helium and gallium focused ion beams. *Journal of Applied Physics* **109.7**, 07D504 (2011).
2. You, L. *et al.* Co/Ni multilayers with perpendicular anisotropy for spintronic device applications. *Applied Physics Letters* **100.17**, 172411 (2012).
